# Evaluation of Dissolved Organic Carbon as a Soil Quality Indicator in National Monitoring Schemes

**DOI:** 10.1371/journal.pone.0090882

**Published:** 2014-03-14

**Authors:** David L. Jones, Paul Simfukwe, Paul W. Hill, Robert T. E. Mills, Bridget A. Emmett

**Affiliations:** 1 School of the Environment, Natural Resources & Geography, Bangor University, Bangor, Gwynedd, United Kingdom; 2 Ecole Polytechnique Fédérale de Lausanne (EPFL), School of Architecture, Civil and Environmental Engineering (ENAC), Laboratory of Ecological Systems (ECOS), Lausanne, Switzerland; 3 Centre for Ecology and Hydrology, Environment Centre Wales, Bangor, Gwynedd, United Kingdom; Tennessee State University, United States of America

## Abstract

**Background:**

Monitoring the properties of dissolved organic carbon (DOC) in soil water is frequently used to evaluate changes in soil quality and to explain shifts in freshwater ecosystem functioning.

**Methods:**

Using >700 individual soils (0–15 cm) collected from a 209,331 km^2^ area we evaluated the relationship between soil classification (7 major soil types) or vegetation cover (8 dominant classes, e.g. cropland, grassland, forest) and the absorbance properties (254 and 400 nm), DOC quantity and quality (SUVA, total soluble phenolics) of soil water.

**Results:**

Overall, a good correlation (*r* ^2^ = 0.58) was apparent between soil water absorbance and DOC concentration across the diverse range of soil types tested. In contrast, both DOC and the absorbance properties of soil water provided a poor predictor of SUVA or soluble phenolics which we used as a measure of humic substance concentration. Significant overlap in the measured ranges for UV absorbance, DOC, phenolic content and especially SUVA of soil water were apparent between the 8 vegetation and 7 soil classes. A number of significant differences, however, were apparent within these populations with total soluble phenolics giving the greatest statistical separation between both soil and vegetation groups.

**Conclusions:**

We conclude that the quality of DOC rather than its quantity provides a more useful measure of soil quality in large scale surveys.

## Introduction

Measurement of dissolved organic matter (DOM) concentrations have provided a sensitive indicator for assessing large scale geographical and temporal (e.g. decadal) changes in water quality [1]. In contrast, while DOM is frequently measured in soil studies, it has rarely been used for evaluating changes in soil quality in large scale (e.g. national) monitoring programmes [2]. To some extent, the DOM concentration of freshwaters should reflect that of the surrounding catchment area suggesting that an assessment of soil DOM might provide a sensitive soil quality indicator. This would also match with studies investigating the progressive increase in DOM within rivers, a widespread trend observed in both Europe and North America [3]–[4]. An investigation of the spatial or temporal changes in soil DOM within different soil types could therefore prove useful for explaining these trends in catchments containing a complex mixture of soils. One potential drawback, however, is that determination of DOM in soil can be very time consuming and expensive when large numbers of samples need to be processed. One potential solution is to use the spectral properties of soil water which provides a rapid proxy for estimating dissolved organic C (DOC) concentration [5]–[10]. For a single geographical location, strong relationships are often apparent between DOC concentration and UV absorbance [11]–[13]. However, these relationships are often non-linear and the mathematical function describing the relationship can vary with season or changes in hydrological flow patterns [14]–[16]. Further, whilst differences in the quality and quantity of DOC are frequently reported for contrasting soil and vegetation types, whether these dissimilarities remain valid over large geographical scales remains uncertain. In this context, we undertook a national large scale survey of the quality and quantity of DOM across Great Britain to evaluate its potential for evaluating changes to soil quality in national soil quality monitoring programmes. Our first aim was to critically evaluate the relationship between direct and indirect methods for DOC quantification. Secondly, we aimed to evaluate whether different soil classes and vegetation cover types possessed unique DOM signatures in terms of both quantity and quality and whether these might be useful for explaining changes in DOM occurring at a national scale.

## Methods

### Soil Sampling

To encompass all the major soil and land use types, a total of 702 soil samples were collected throughout Great Britain in June-July, 2007 (area 209,331 km^2^; ca. 300 km^2^ sample^−1^) as part of the Centre for Ecology and Hydrology Countryside Survey (CS) [17]. Samples were selected, based on a stratified random sample of 1 km squares at gridpoints on a 15 km grid using the Institute of Terrestrial Ecology (ITE) Land Classification as the basis of the stratification (Fig. S1). At each grid intersection, a 1 km^2^ sample area was selected. Within the 1 km^2^ sample area, a 5×5 m^2^ plot was randomly located and replicate 15 cm long ×4 cm diameter soil cores were collected. Topsoils (0–15 cm) were only sampled to reflect past and current (1978-present) standard practice in UK national monitoring [18]–[20]. This depth was originally selected by the UK Government's Department for Environment, Food and Rural Affairs as it was hypothesized that it would show the greatest change in response to environmental perturbation (including changes in land use, climate, agronomic management or atmospheric deposition). The soil horizons sampled included H, O and A horizons with *a*, *e*, *i*, *h*, *g*, *k* and *p* sub-designations [21]. Across all land use categories, the dominant eight soil groupings (% of total) were: Brown soils (31%), Podzolic soils (15%), Surface water (SW) gley soils (18%), Peat soils (13%), Groundwater (GW) gley soils (12%), Lithomorphic soils (8%), and Pelosol soils (3%) [22]. The FAO World Reference Base Classification equivalent categories for these soil groups are presented in Table S1 and their major chemical, physical and biological properties presented in Table 1. Vegetation cover at each sampling point was classified into eight aggregated vegetation classes (AVC) with the following groupings (% of total): Cropland (15%), Tall grass and herbs (4%), Fertile grassland (19%), Infertile grassland (21%), Lowland woodland (3%), Upland woodland (8%), Moorland grassland mosaic (11%), and Heathland and bog (19%) [17]. Aggregate vegetation classes were derived by cluster analysis of the mean DECORANA scores for 100 smaller classes obtained by TWINSPAN analysis of plant species data in each sample plots [37]. Further descriptions of the vegetation types can be found in Table S2.

**Table 1:** Properties of the major soil groups.

Soil group	Soil pH	Soil organic matter (%)	C-to-N ratio	Olsen P (mg kg^−1^)	Bulk density (g cm^−3^)	Exchangeable Ca (mmol kg^−1^)	Exchangeable Al (mmol kg^−1^)	Soil respiration (mg C cm^−3^ h^−1^)	Soil respiration (g C g SOC^−1^ h^−1^)
Pelosol	6.8±1.4^a^	7.3±3.3^cd^	10.7±0.8^de^	28±22^abc^	1.10±0.21^a^	62±40^a^	1.3±4.8a^b^	10.2±26.4^bc^	4.7±3.4^a^
Brown	6.5±1.2^a^	10.0±12.5^d^	12.2±4.1^e^	32±27^abc^	1.04±0.33^a^	39±27^a^	2.1±4.8^b^	8.6±11.0^c^	7.3±5.6^a^
Groundwater gley	6.6±1.2^a^	10.9±13.5^d^	13.4±4.4^cde^	32±34^abc^	0.98±0.36^a^	42±31^a^	2.3±5.4^ab^	13.2±24.1^bc^	8.1±6.4^a^
Surface water gley	5.9±1.3^a^	24.9±30.0^bc^	14.2±5.5^cd^	25±28^bc^	0.78±0.43^b^	54±49^a^	6.3±14.6^ab^	14.9±19.2^bc^	6.6±5.0^ab^
Podzolic	4.9±0.9^b^	28.8±27.8^b^	17.9±7.0^b^	21±20^c^	0.57±0.33^c^	20±29^b^	7.1±8.9a^b^	17.9±32.9^bc^	5.1±3.9^b^
Lithomorphic	6.0±1.4^a^	35.1±32.8^b^	16.2±6.4^bc^	41±69^ab^	0.56±0.38^c^	42±39^a^	3.4±5.6^ab^	20.8±33.0^b^	6.4±5.6^ab^
Peat	4.6±0.6^b^	75.9±27.9^a^	24.1±8.5^a^	44±51^a^	0.20±0.21^d^	46±50^a^	9.6±38.0^a^	43.1±37.1^a^	4.5±3.8^b^
ANOVA	***	***	***	**	***	***	**	***	***

Values are expressed on a dry weight basis and represent mean ± SD.

** and *** indicate significant differences between soil groups at the *P*<0.01 and *P*<0.001 levels respectively. Superscript letters indicate significant between soil groups at the *P*<0.05 level.

### Soil Water Collection and Analysis

Soil water was obtained by adding artificial rainwater to the intact soil columns and collecting the leachate as detailed in [23]–[24]. The absorbance of the soil leachate water was measured at 254 and 400 nm on a Synergy 96 well plate spectrophotometer (BioTek Instruments Inc., Winooski, VT) using Falcon flat-bottom UV well plates (BD Biosciences, Franklin Lakes, NJ). DOC concentrations were measured with a TOC-V analyser (Shimadzu Corp., Kyoto, Japan). Total dissolved phenolics and tannins were assayed colorimetrically using the Folin-Ciocalteu reagent (F9252; Sigma-Aldrich Inc.) according to [25] using gallic acid as a standard. Specific ultraviolet absorbance (SUVA) was calculated by dividing the absorbance at 254 nm (cm^−1^) by the DOC concentration (mg l^−1^).

### Background Soil Analysis

Background soil characteristics were measured on replicate cores matching those used in the analysis above. After collection from the field, the soil was extruded from the core, roots and stones removed and the soil homogenised and dried (105 °C). Total soil C and N were analysed on an Elementar Vario-EL elemental analyser (Elementar Analysensysteme GmbH, Hanau, Germany) using the UKAS accredited method SOP3102 [23]. Bulk density was calculated as mass/volume after the removal of stones (>2 mm) and accounting for their volume [23]. Soil organic matter was determined by loss-on-ignition (LOI) by first drying soil (10 g) at 105°C and then measuring the mass loss after further heating at 375 °C for 16 h. Available soil P was measured using the Olsen method whereby 5 g of soil was extracted with 100 ml of 0.5 M sodium bicarbonate (pH 8.5). The P in the extract was determined colorimetrically using the molybdate blue method (880 nm) using a continuous flow analyser [23]. Soil pH was measured by equilibrating 10 g of field-moist soil with 25 ml of deionised water. Exchangeable Ca and Al were determined by shaking 5 g of soil with 25 ml of 1.0 M NH_4_Cl (250 rev min^−1^, 60 min). Subsequently, the extracts were centrifuged (5000 *g*, 10 min) and the supernatant recovered for analysis. Ca in the extracts was determined by atomic absorption spectrometry on a Perkin Elmer Analyst 400 Atomic Absorption Spectrometer (PerkinElmer, Waltham, MA, USA). The extracts were diluted with LaCl_3_ (0.5% w/v) prior to Ca determination. Al concentration in the extracts was determined using the modified catechol violet method [35]. The absorbance of the solution was measured at 580 nm using a PowerWave XS scanning microplate spectrophotometer (BioTek Instrument, Winooski, VT). Basal soil respiration was determined on one replicate core. The cores were wet to field capacity as described previously, placed in a sealed chamber (1250 cm^3^ head space). The soils were then incubated at 10°C (average UK air temperature) for 1 h (at which linearity was known to be established following testing on selected cores which covered the range of soil types sampled). Subsequently, the head space gas was analysed for CO_2_ concentration using a Clarus 500 Gas Chromatograph (PerkinElmer). SR was determined as the change in CO_2_ concentration before and after incubation corrected for soil dry weight and soil organic matter content.

### Statistical Analysis

Linear and stepwise regression analysis was undertaken using Minitab v16 (Minitab Inc., State College, PA). When the solution DOC, absorbance, soluble phenolic and SUVA values were grouped according to soil and vegetation type the data failed normality testing (Shapiro-Wilk). Consequently, the data were log transformed, normality verified and an ANOVA performed with Tukey-pairwise comparisons (*P*<0.05 cut-off) using Minitab v16.

## Results

### Soil Water Properties

**Figure 1:**
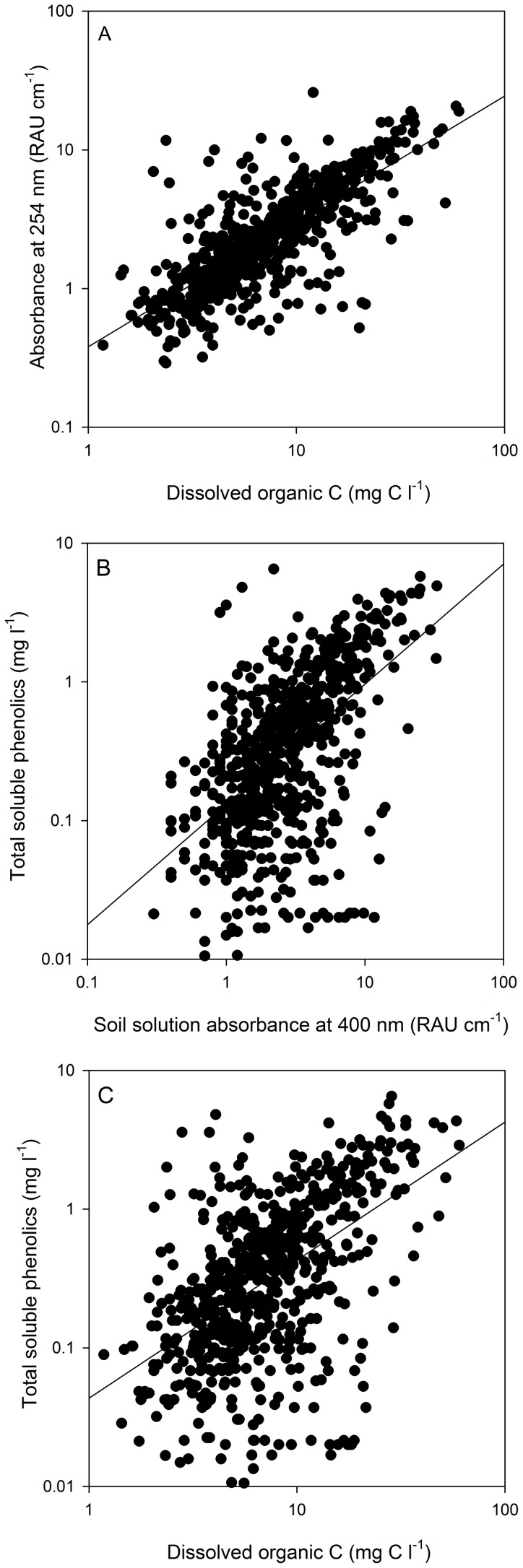
Relationship between the absorbance of soil water from 702 individual soils measured in the UV (254 nm) range and DOC concentration (Panel A), the relationship between total soluble phenolics and either solution absorbance at 400 nm (Panel B) or DOC concentration (Panel C). The lines are linear regression for all the data in the plots (Panel A, *r* ^2^ = 0.579; y = 0.387+0.301x; Panel B, *r* ^2^ = 0.432; y = 0.12+0.139x; Panel C, *r* ^2^ = 0.375; y = 0.025+0.068x).

The relationship between the absorbance of soil water in the UV (254 nm) and visible (400 nm) range across 702 individual sites showed a strong linear correlation (*r* ^2^ = 0.931; *P*<0.001; Fig. S2). A strong positive correlation was also observed between the DOC concentration in soil water and both absorbance at 254 nm (*r* ^2^ = 0.58; *P*<0.001; Fig. 1) and 400 nm (*r* ^2^ = 0.47; *P*<0.001). The prediction of DOC concentration using both absorbance values in a stepwise regression model did not result in a significantly better fit (*r* ^2^ = 0.61). In contrast, the absorbance characteristics of soil water proved to be a less reliable predictor of total soluble phenolics (254 nm *r* ^2^ = 0.41; *P*<0.001; 400 nm *r* ^2^ = 0.43; *P*<0.001; Fig. 1). Similarly, DOC concentration either with or without inclusion of spectral properties into the regression model, proved to be the least reliable predictor of total soluble phenolics (*r* ^2^ = 0.38; *P*<0.001). SUVA proved to be the worst predictor of total soluble phenolics with either linear (*r* ^2^ = 0.13) or non-linear models (data not presented).

### Is Soil or Vegetation a Better Predictor of DOC Quantity and Quality?

**Figure 2:**
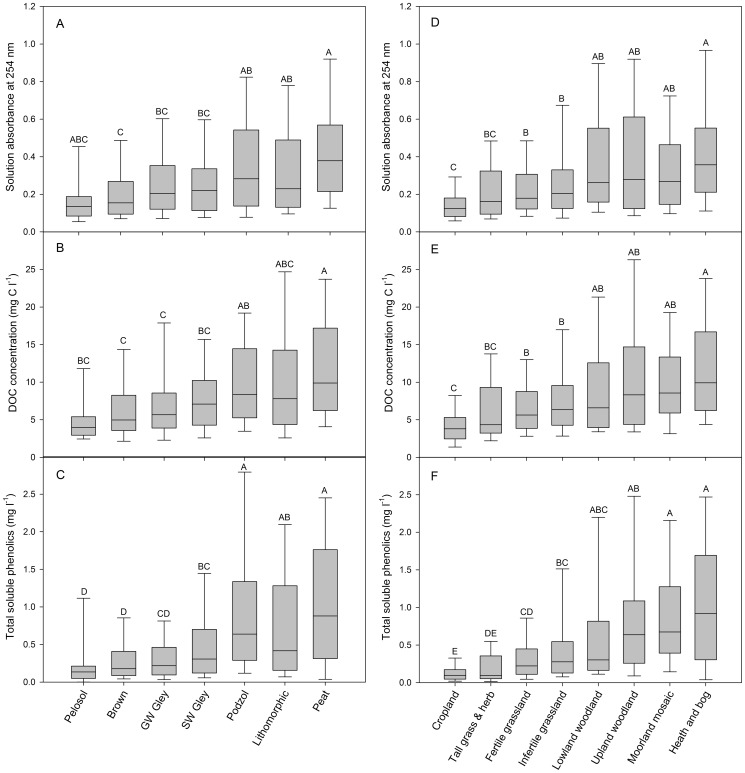
Box plots showing the influence of soil class (panels A–C) and vegetation cover type (panels D–F) on the absorbance of soil solutions at 254 nm, their DOC and total soluble phenolic concentration from 702 individual soils sampled as part of a nationwide soil quality assessment. The boundary of the box closest to zero indicates the 25^th^ percentile, the line within the box marks the median, and the boundary of the box farthest from zero indicates the 75^th^ percentile. Whiskers (error bars) above and below the box indicate the 90^th^ and 10^th^ percentiles respectively. Different letters indicate significant difference between individual groups (*P*<0.05). Soils are ranked in organic matter (OM) content from left (low OM) to right (high OM) while vegetation is broadly ranked according to agricultural productivity.

The quantity and quality of DOC as a function of different soil class and vegetation types is shown in Figure 2. Overall, there was a large similarity in the measured ranges of DOC, absorbance at 254 nm and soluble phenolics between the different groupings for both soil and vegetation. Despite this, ANOVA revealed significant differences between groups (all *P*<0.001) with response gradients apparent when the groups were ranked according to either organic matter content (soils) or an intensification/altitude scale (vegetation)(Fig. 2). Of the parameters measured, soluble phenolic content gave the greatest separation between groups. In contrast, SUVA revealed no significant differences between groupings for both soil (*P* = 0.483) and vegetation (*P* = 0.819; Fig. S3).

## Discussion

### Method of Soil Sampling

The sampling undertaken here formed part of a nationwide monitoring programme which is carried out each decade to assess changes in soil and water quality, landscape features and aquatic and terrestrial biodiversity [19], [26]. Whilst the sampling depth (0–15 cm) is highly suited to agricultural soils which show a high degree of vertical homogeneity (due to cultivation), we acknowledge that the sampling regime may be less suited to highly stratified soils [27]. In these horizonated soils, differential amounts of O, E, A and possibly B horizons could be sampled by coring from 0–15 cm, depending on their thickness. This has led to many studies, particularly in forests, where soil quality is measured on organic and mineral horizons separately [27]–[28]. In our case, the historical legacy is such that the sampling protocols will not be changed for the foreseeable future (on scientific, socio-political and economic grounds) and therefore our results should be viewed in the context of this.

### DOC and Soil Quality Assessment

Overall, our results show that in large geographical scale soil quality assessments across a broad range of vegetation and soil types, soil water DOC concentrations can be rapidly and cheaply estimated from their spectral properties. This is particularly relevant as DOC in itself can be used to predict the movement of both organic and inorganic pollutants in soil and is often seen as a pollutant itself when entering freshwaters [9], [29]–[30]. Disappointingly, however, we found that all soil and vegetation types had a wide variability in their DOC concentrations. This indicates that the quantity of DOC may not represent a sensitive indicator for monitoring the stability of ecosystems when faced with anthropogenic perturbation (e.g. land use change). This is in contrast to [24] who found different rates of N mineralization (NH_4_^+^+NO_3_^−^) in the same soils alongside other soil quality indicators as shown in Table 1. While SUVA is frequently used for assessing the quality of DOC in freshwaters [30], our results indicated that it was incapable of separating between land uses and soil classes over a wide geographical range and is therefore probably unsuited to large scale soil quality assessments. In contrast, total soluble phenols gave the best separation between ecosystem types. This was a surprising result as we had assumed that SUVA and soluble phenols would be highly correlated. This disagreement could be due to interference in both the determination of total phenolics (e.g. SO_2_, DOC; [31]–[32]) or SUVA (e.g. pH, Fe^3+^; [12]). However, more likely it is due to the natural variability in the total phenol content of humic substances originating from different soil classes and vegetation cover types [33]–[34]. As expected, the quality and quantity of DOC were dependent on both vegetation and soil type. This is to some extent expected considering that certain soil types favour certain vegetation covers, however, it does indicate that 2-way stratification by soil and vegetation type may prove more useful for revealing unique DOC signatures.

With respect to the measurement of DOC quality, we acknowledge that recent methodological advancements are increasing our potential to characterise the many thousands of compounds that comprise DOC [36]. It is highly likely that these new analytical approaches will reveal compounds that are specific to different functional soil or plant types (e.g. keystone compounds) improving our capacity to use DOC as a soil quality indicator. In addition, this analysis may also directly support the interpretation of temporal changes in other soil quality indicators (e.g. soil organic matter quality and quantity, soil biodiversity).

## Supporting Information

Figure S1 **Map of the UK showing the individual soil sampling locations used in the study.** The total land area is 209,331 km2.
(TIF)

Figure S2 **Relationship between the absorbance of soil water from 702 individual soils measured in either the UV (254 nm) or visible (400 nm) range.** The line is a linear regression for all the data in the plot (*r* ^2^ = 0.931; y = −0.01+0.123x).
(TIF)

Figure S3 **Box plots showing the influence of soil type (panel A) and vegetation cover (panel B) on the specific UV absorbance (SUVA) values from 702 individual soils sampled as part of a nationwide soil quality assessment.** The boundary of the box closest to zero indicates the 25^th^ percentile, the line within the box marks the median, and the boundary of the box farthest from zero indicates the 75^th^ percentile. Whiskers (error bars) above and below the box indicate the 90^th^ and 10^th^ percentiles respectively. No significant differences were apparent between treatments.
(TIF)

Table S1 **Comparable classification of the UK soil groups with those in the FAO World Reference Base Classification (WRB, 2006).**
(DOCX)

Table S2 **Summary descriptions of the eight aggregate vegetation classes represented. Table adapted from Smart et al. (2003).**
(DOCX)
