# Supplementary figures and images for: Evaluation of Dissolved Organic Carbon as a Soil Quality Indicator in National Monitoring Schemes
Source: PLoS One. 2014 Mar 14;9(3):e90882. doi: 10.1371/journal.pone.0090882 (PMC3954595; doi:10.1371/journal.pone.0090882)

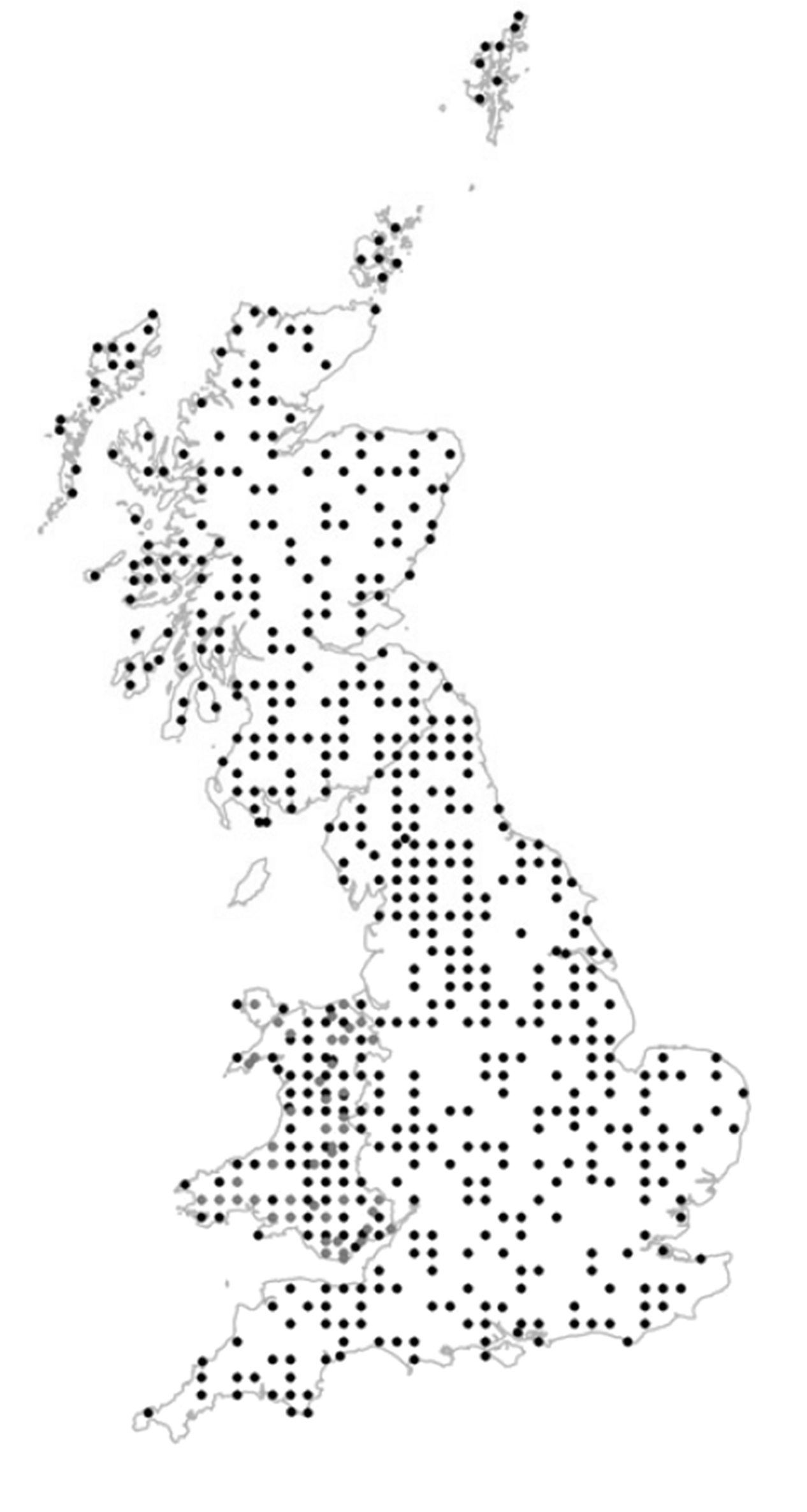

Supplement: Figure S1 — Map of the UK showing the individual soil sampling locations used in the study. The total land area is 209,331 km2. (TIF) [file pone.0090882.s001.tif]

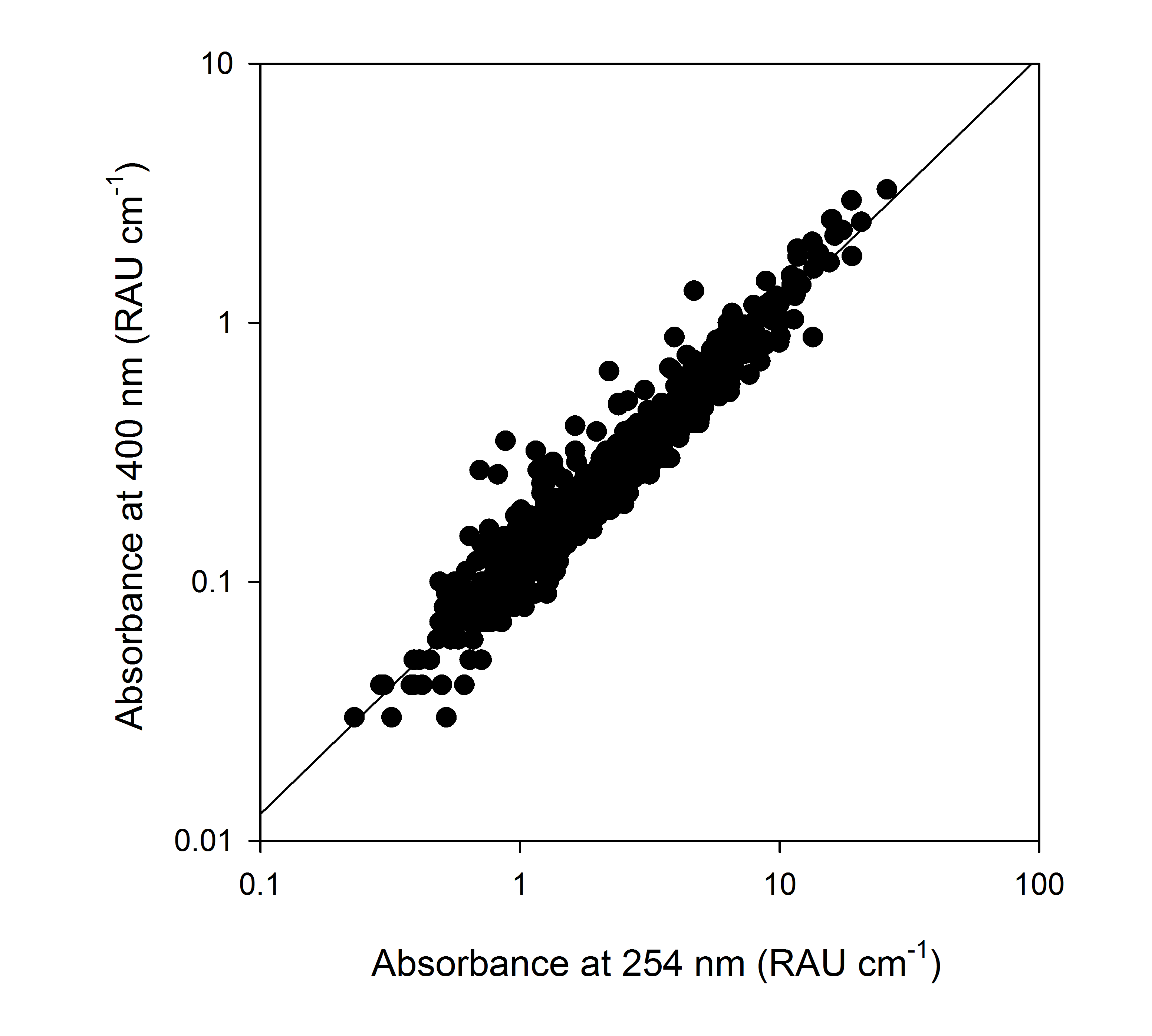

Supplement: Figure S2 — Relationship between the absorbance of soil water from 702 individual soils measured in either the UV (254 nm) or visible (400 nm) range. The line is a linear regression for all the data in the plot (r 2 = 0.931; y = −0.01+0.123x). (TIF) [file pone.0090882.s002.tif]
