# Supplementary material for: Evaluation of Dissolved Organic Carbon as a Soil Quality Indicator in National Monitoring Schemes
Source: PLoS One. 2014 Mar 14;9(3):e90882. doi: 10.1371/journal.pone.0090882 (PMC3954595; doi:10.1371/journal.pone.0090882)
Supplement: Table S1 — Comparable classification of the UK soil groups with those in the FAO World Reference Base Classification (WRB, 2006). (DOCX) [file pone.0090882.s004.docx]

Table S1. Comparable classification of the UK soil groups with those in the FAO World Reference Base Classification (WRB, 2006).

| **Major UK soil group** | **World Reference Base** |
| --- | --- |
| Brown | Mainly Cambisols with some Luvisols, Acrisols |
| Lithomorphic | Leptosols and some Regosols |
| Surface and groundwater gleys | Mainly Gleysols, Planosols and some Fluvisols/Luvisols |
| Podzolic | Podzols |
| Peat | Histosols |
| Pelosol | Vertisols |

The soils at the sampling points were classified during two previous surveys (1978 and 1990) using the British soil classification system of Avery (1973, 1980). In 1978, pits were dug at each site of the selected plots in the 1 km squares; soils were sampled and classified to the sub group level. In 1990, the soils were surveyed and mapped at 1:25 000 using the Soil Survey Technical Monograph No.14 (Avery, 1980) by the Macaulay Land Use Research Institute in Scotland and the Land Research Centre (now the National Soil Research Institute) in England and Wales. The major soil groups used here were a product of a rigorous comparison process between 1978 data and 1990 maps. The description was derived and allocated manually and therefore the product was a more accurate classification than either the 1978 or the 1990 classifications taken in isolation.

**References**

Avery BW (1973) Soil Classification in the Soil Survey of England and Wales. J. Soil Sci. 24: 324-38.

Avery BW (1980) Soil classification for England and Wales. Soil Survey Technical Monograph No.14, Harpenden, UK.

WRB (2006) World Reference Base for Soil Resources 2006. Report 103. Food and Agriculture Organization of the United Nations, Rome, Italy.
