# Supplementary material for: Evaluation of Dissolved Organic Carbon as a Soil Quality Indicator in National Monitoring Schemes
Source: PLoS One. 2014 Mar 14;9(3):e90882. doi: 10.1371/journal.pone.0090882 (PMC3954595; doi:10.1371/journal.pone.0090882)
Supplement: Table S2 — Summary descriptions of the eight aggregate vegetation classes represented. Table adapted from Smart et al. (2003). (DOCX) [file pone.0090882.s005.docx]

Table S2. Summary descriptions of the eight aggregate vegetation classes represented. Table adapted from Smart et al. (2003).

| **Aggregate vegetation classes** | **Description** |
| --- | --- |
| Crops/weeds | Weedy communities of cultivated and disturbed ground, including species poor arable and horticultural crops. |
| Tall grassland/herb | Less intensively managed tall herbaceous vegetation typical of field edges, roadside verges, stream sides and hedge bottoms. |
| Fertile grass | Improved or semi improved grasslands. Often intensively managed agricultural swards with moderate to high abundance of perennial rye grass. |
| Infertile grass | Less productive unimproved and often species rich grasslands in a wide range of wet to dry and acidic to basic situations. |
| Lowland wooded | Vegetation dominated by shrubs and trees in neutral or basic situations, generally in lowland Britain. Includes many hedgerows. |
| Upland wooded | Vegetation of broad leafed and conifer woodland often in more acidic situations, generally in upland Britain. |
| Moorland grass/mosaic | Extensive, often unenclosed and sheep grazed hill pastures. |
| Heath/bog | Vegetation dominated by heathers. Included drier heaths as well as bog. Mostly in the uplands. |

**Reference**

Smart SM, Robertson JC, Shield EJ, Van De Poll HM (2003) Locating eutrophication effects across British vegetation between 1990 and 1998. Glob. Change Biol. 9: 1763-1774.
